# Protective effect of Xuebijing injection on paraquat-induced pulmonary injury via down-regulating the expression of p38 MAPK in rats

**DOI:** 10.1186/1472-6882-14-498

**Published:** 2014-12-16

**Authors:** Ming-wei Liu, Mei-xian Su, Wei Zhang, Yan-qiong Wang, Mei Chen, Li Wang, Chuan-yun Qian

**Affiliations:** Department of Emergency, The First Hospital Affiliated To Kunming Medical University, 295 Xichang Road, Wu Hua District, Kunming, 650032 China; Intensive Care Unit, The Second Hospital Affiliated To Kunming Medical University, 1 Mayuan, Wu Hua District, Kunming, 650106 China; Department of Anesthesiology, The First Hospital Affiliated To Kunming Medical University, 295 Xichang Road, Wu Hua District, Kunming, 650032 China; Department of Respiratory Medicine, The Yan-an Hospital Affiliated To Kunming Medical University, 245 Renmin Eastern Road, Pan Long District, Kunming, 650051 China

**Keywords:** Xuebijing, Paraquat, Acute lung injury, p38 MAPK, NF-κB65, Rat

## Abstract

**Background:**

Exposure to paraquat results in acute lung injury. A systemic inflammatory response has been widely established as a contributor to paraquat-induced acute lung injury. Recent studies have reported that consumption of Xuebijing prevents inflammatory response-induced diseases. This study investigated whether consumption of Xuebijing protected rats against paraquat-induced acute lung injury.

**Methods:**

Adult male Sprague Dawley rats were randomly divided into four groups: control group; paraquat group; paraquat + Xuebijing group; and paraquat + dexamethasone group. Rats in the paraquat, paraquat + Xuebijing and paraquat + dexamethasone groups were intraperitoneally injected with paraquat (30 mg/kg) or administered paraquat and Xuebijing at 8 mL/kg or dexamethasone at 5 mg/kg, respectively, via an injection into the tail vein. Lung p38 MAPK, NF-κB65, IkB, p-IκB-α, HIF-1α, Nrf2 and TGF-β1 expression were essayed using western blotting. IL-6, TNF-α, IL-1β, IL-10, TGF-β1 and PIIIP were measured using ELISA. ROS, oxidised glutathione and glutathione activity were measured.

**Results:**

After inducing acute lung injury with paraquat for 24 h, Xuebijing was observed to block lung p-p38 MAPK, NF-κB65, HIF-1α, p-IκB-α and TGF-β1 expression, and increased Nrf2 and IkB expression. The numbers of neutrophils and lymphocytes and total number of cells were significantly lower in the Xuebijing group compared with the control group. IL-6, TNF-α, IL-1β, TGF-β1 and PIIIP levels were significantly decreased in the Xuebijing group. ROS and oxidised glutathione activity were markedly inhibited by Xuebijing. Histological evaluation showed attenuation of the effects of Xuebijing on paraquat-induced lung injury. Compared with the paraquat + dexamethasone group, the Xuebijing + paraquat group showed no significant differences.

**Conclusions:**

Inhibiting the expression of p38 MAPK and NF-κB65 was crucial for the protective effects of Xuebijing on paraquat-induced acute lung injury. The findings suggest that Xuebijing could effectively ameliorate paraquat-induced acute lung injury in rats. Xuebijing was as effective as dexamethasone at improving paraquat-induced lung injury by regulating lung inflammation, lung function and oxidative stress responses.

## Background

Paraquat (1,1-dimethyl-4,4-bipyridilium dichloride) is a widely used contact and nonselective quaternary nitrogen herbicide. Since its first introduction into agricultural use in 1962, it has caused thousands of human deaths, either by accidental or voluntary ingestion. The toxicity of paraquat is based on its induction of redox cycling, which results in oxidative stress-related cell death and inflammation. Because of its selective accumulation in the lungs, it causes severe lung injury, manifesting in oedema, haemorrhage, interstitial inflammation, and progressive fibrosis [1, 2]. Many studies have suggested that the mechanisms of paraquat-induced lung injury are mainly associated with the systemic inflammatory response [3, 4]. Histologically, acute lung injury (ALI) in humans is characterised by a severe acute inflammatory response in the lungs and neutrophilic alveolitis [5]. The physiological hallmark of acute respiratory distress syndrome is disruption of the alveolar-capillary membrane barrier, which results in the development of noncardiogenic pulmonary oedema in which a proteinaceous exudate floods the alveolar spaces, impairs gas exchange and precipitates respiratory failure [6, 7]. ALI can result in persistent respiratory failure and prolonged dependence on mechanical ventilation, thereby increasing susceptibility to multiorgan dysfunction and mortality [8]. Despite extensive investigation aimed at early diagnostic and pathogenic factors of ALI, current management is mainly supportive because specific therapies have not been identified [9–11]. Animal models focused on ALI pathogenesis have yielded insights into mechanisms that initiate injury; however, little is known about the potential determinants of treatment [12, 13]. Thus, new strategies are still required to achieve effective treatment of ALI, which might ultimately aid in clinical therapy for ALI patients.

Numerous basic and clinical studies have demonstrated that overexpression of TNF-α and IL-1ß can induce ALI [14]. In addition, cytokine production is associated with activation of the p38-mitogen activated protein kinase (p38 MAPK) pathway [15]. Cytokine production is also regulated by p38 MAPK. Inactivated p38 MAPK is normally located in the cytoplasm and is translocated into nuclei following activation, where it activates the activator protein-1 by phosphorylating myocyte-specific enhancer factor-2C, activating transcription factor-2, and E twenty-six-like transcription factor-1, thereby regulating the production of TNF-α and IL-1ß.

Xuebijing is a Chinese herbal compound preparation mainly consisting of Chuanxiong (Rhizoma Chuanxiong), Chishao (Radix Paeoniae Rubra), Danshen (Radix Salviae Miltiorrhiae), and Honghua (Flos Carthami). Xuebijing can clear heat, cool blood, promote gas and blood circulation, remove toxic substances, and relieve pain [16]. Furthermore, Xuebijing has been used to treat systemic inflammatory response syndrome, pyemia, and multiple organ dysfunction syndrome [17].

In the present study, we hypothesised that Xuebijing could reduce inflammatory-induced lung injury following paraquat poisoning. We examined the expression of cytokines and p38 MAPK in the lung following treatment with Xuebijing in a rat model of paraquat–induced lung injury.

## Methods

### Drugs

Xuebijing injections were obtained from Tianjin Chase Sun Pharmaceutical Co., Ltd. (No. Z20040033; Tianjin, China), and consisted of Chuanxiong, Chishao, Danshen and Honghua. Chuanxiong, Chishao, Danshen and Honghua were obtained from Prof. Li Shixia of Central South University and deposited at the Pharmacy Centre.

### Reagents

Rabbit anti-mouse NF-κB65 and p38 MAPK antibodies were obtained from Santa Cruz Biotechnology (Santa Cruz, CA, USA). Mouse TNF-α, IL-1β and IL-6 ELISA kits were purchased from Quantikine, R&D Systems (Minneapolis, MN, USA). The Griess reagent nitric oxide assay kit was purchased from Beyotime Biotech (Jiangsu, China). The mouse IL-10 ELISA kit was obtained from Bender MedSystems (Vienna, Austria). Paraquat was purchased from Sigma.

### Main instruments

An Olympus inverted microscope (Olympus, Tokyo, Japan), low-temperature refrigerated centrifuge (Eppendorf, Germany), clean bench (Suzhou Purification Equipment Co., Ltd.), continuous wavelength microplate reader (Bio-Rad Laboratories Inc., Hercules, CA, USA), electronic balance (Sartorius Instrument & System Engineering Co., Ltd, Beijing, China), haemocytometer (Qiujing Biochemical Instrument Factory, Shanghai, China), AE31 inverted phase contrast microscope (Motic), and electric homogeniser (Kemi Instrument & Meter Co., Ltd. Zhenjiang, China) were used in this study.

### Experimental animals

Male Sprague Dawley rats were purchased from Kunming Medical University Laboratory Animal Center (Kunming, China). All rats were housed at the Kunming Medical University Animal Care Facility and were maintained in pathogen-free conditions. Rats were 8–9 weeks of age at the initiation of the experiment and were maintained on standard laboratory chow and water was provided *ad libitum*. All experiments were approved and performed according to the guidelines of the Animal Care Committee of Kunming Medical University.

### Preparation of Xuebijing from Chuanxiong, Chishao, Danshen and Honghua

We prepared Xuebijing as previously described in the literature [18]. The appropriate amount of dried Chuanxiong, Chishao, Danshen and Honghua was weighed and twice backflow extractions were performed using chloroform:methanol (2:1) at 50°C in a water bath for 2 h at each time point. The liquid was discarded, and ultrasonic extraction with the residue with 80% ethanol was performed three times (20 min each) and filtered. The filtrate was discarded for ultrasonic extraction with water three times (20 min each) and filtered. To lower the temperature of the concentration of the combined filtrate, 95% ethanol was added, and the solution was allowed to stand at a low temperature for 24 h. To obtain Xuebijing, several washes were performed and the solids were extracted and filtered with absolute ethanol and acetone prior to vacuum drying.

### HPLC determination of Xuebijing

Analyses were performed using a liquid chromatography system (Waters, Milford, MA, USA) with 515 pumps, equipped with an online degasser, a Waters pump control module, an autosampler 717, a Waters 2996 photodiode array detector, and Waters Empower software. Separation was performed using a Supelcosil LC-8-DB column (250 × 4.6 mm i.d.; 5 μm pore size) with a guard column (40 × 4.6 mm i.d.) packed with the same material. The column was maintained at 25°C throughout the analysis, and detection was performed at 254 nm. Elution was performed at a flow rate of 0.8 mL/min with water as solvent A and methanol as solvent B. In addition, an isocratic elution from 0–10 min with 90% of A followed by a gradient elution from 10–15 min with 90–85% of A, 15–30 min with 85–70% of A, 30–37 min with 70–90% of A, and isocratic from 37–45 min with 90% of A was performed. A stock solution of Xuebijing (1 mg/mL) was prepared in methanol and analysis was performed under the same working conditions. Each analysis was repeated three times, and the respective retention times were averaged. Peak identification in HPLC analysis was performed by comparing the retention time with the reference standard. Quantification of the compounds was achieved using the calibration plot of the standard solution.

The concentrations for the standard used for the calibration curve ranged from 1.0 μg to 5.0 μg for Xuebijing. Each run was repeated three times.

**Figure 1:**
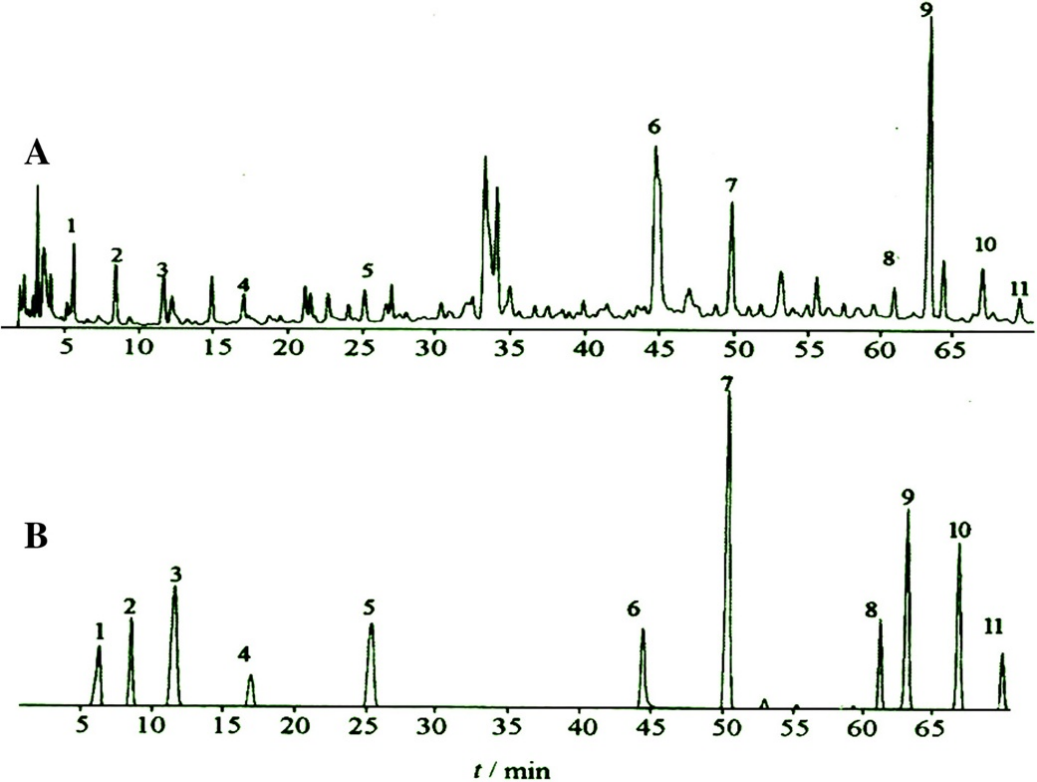
**Chromatogram of the chemical reference substances and sample. A**: Chromatogram of the chemical reference substances. **B**: Chromatogram showing the following samples: 1, uridine; 2, gallic acid; 3, guanosine; 4, danshensu sodium; 5, paeoniflorin; 6, protocatechuic aldehyde; 7, ferulic acid; 8, salvianolic acid B; 9, safflor yellow A; 10, senkyunolide; and 11, senkyunolide.

Chromatography was performed at room temperature with a flow rate of 1.0 mL/min, and 10 μL of volume was analysed. Analysis (Figure 1) showed that 1 L of the Xuebijing injection contained 38.61 mg uridine, 11.98 mg gallic acid, 27.04 mg guanosine, 48.33 mg danshensu sodium, 7.15 mg paeoniflorin, 19.6 mg protocatechuic aldehyde, 37.40 mg ferulic acid, 29.82 mg salvianolic acid B, 48.66 mg safflor yellow A, 53.15 mg senkyunolide I, and 19.29 mg senkyunolide.

### Animal groupings and drug administration

Briefly, rats were randomly assigned into one of four groups: the saline control group, paraquat group, Xuebijing + paraquat group and dexamethasone + paraquat group (*n* = 20 for each group). From previous experiments and reports [2, 16], ALI was induced by an intraperitoneal paraquat injection (30 mg/kg) and left untreated for 1 h. Subsequently, rats were given either Xuebijing 8 mL/kg, dexamethasone 5 mg/kg or 300 μl of normal saline via injection into the tail vein. Three to five rats from each group were anaesthetised and killed at 48-h post-injection of paraquat for cytokine concentration measurements, assessment of lung injury, and histology.

### Collection of bronchoalveolar lavage fluid (BALF) and tissue samples

Rats were euthanised and their thoraxes were opened via a midline thoracotomy. Three millilitres of blood was collected from the heart and centrifuged at 2000 × *g* at 4°C for 10 min. The serum was collected and stored at −80°C until further analysis. After euthanising the rats, the trachea was isolated and the right bronchial tube was ligated. BALF was obtained by placing a 20-gauge catheter into the trachea through which 3 mL of cold PBS was flushed back and forth three times. BALF was centrifuged at 3000 × *g* for 20 min at 4°C. The resulting cell pellet was used to determine the total cell count using a counter (Beckman Coulter). A cell smear was made using Wright–Giemsa staining to confirm the percentage of inflammatory cells. The protein concentration of the cell-free BALF from all groups was measured using a Bio-Rad protein assay kit and was used as an indication of endothelial and epithelial permeability. The right middle lung lobes were stored in liquid nitrogen at −80°C until analysis. The right upper lobes were used to quantify the magnitude of pulmonary oedema. The right lower lobes were used for histological evaluation.

### Western blotting

Lung tissues were homogenised in lysis buffer containing protease inhibitors, and protein concentrations were determined using Bradford reagent (Bio-Rad). Samples were loaded onto an SDS-PAGE gel. After electrophoresis at 120 V for 90 min, the separated proteins were transferred onto polyvinylidene difluoride membranes (GE Healthcare, Little Chalfont, United Kingdom) by the wet transfer method (250 mA, 90 min). Nonspecific sites were blocked with 5% non-fat dry milk in Tris-buffered saline with Tween 20 (25 mM Tris, pH 7.5, 150 mM NaCl, 0.1% Tween 20) for 1 h, and the blots were incubated overnight at 4°C with anti-TGF-β1 antibody (Sigma-Aldrich), anti-p38 MAPK antibody (Cell Signaling Technology Inc.), anti-p-p38 MAPK antibody (R&D Systems, Inc.), anti-NF-κB65 antibody (Cell Signaling Technology Inc.), anti-IκB antibody (Cell Signaling Technology Inc.), anti-p-IκB-α antibody (Cell Signaling Technology Inc.), anti-HIF-1α antibody (Cell Signaling Technology Inc.), and anti-Nrf2 antibody (Cell Signaling Technology Inc.). Anti-rabbit or anti-mouse horseradish peroxidase conjugated-IgG antibodies were used to detect binding of the antibodies. The membranes were stripped and reblotted using an anti-actin antibody (Sigma-Aldrich) to normalise the loading of protein in each lane. The binding of specific antibodies was visualised by exposing the membranes to photographic film after treatment with enhanced chemiluminescence system reagents (GE Healthcare).

### Determination of the activation of NF-κB65 using immunohistochemistry

Immunostaining was performed on lung sections after antigen retrieval using Retrievagen A (Zymed, South San Francisco, CA, USA) at 100°C for 20 min, and endogenous peroxidases were quenched with 3% H_2_O_2_. Sections were blocked with 2% BSA in PBS followed by staining with primary anti-NF-κB65 at room temperature for 1 h. Sections were washed, and after application of the secondary antibody (R&D Systems), tissues were developed using Vectastain ABC (Vector Labs, Burlingame, CA, USA) and 3,3′-diaminobenzidine (Vector Labs). Using Image Pro Plus image analysis software (Media Cybernetics, Bethesda, MD, USA), NF-κB65-positive expression in lung tissue was determined and expressed as positive units.

### Determination of cytokines, TGF-β1 and PIIIP in serum using ELISA

Nalgene Nunc MaxiSorp plates were coated with primary antibodies for either IL-6, TNF-α, IL-1β, IL-10, TGF-β1 or PIIIP (R&D Systems) for 1 h at room temperature and washed with PBS and 0.5% Tween 20. After blocking with casein, the samples were added to plates for 1 h at room temperature. Following washing, biotinylated secondary antibodies were applied for 1 h followed by streptavidin-HRP conjugate (Jackson ImmunoResearch, West Grove, PA, USA) diluted at 1:20,000. The reaction was developed with 0.01% tetramethylbenzidine dissolved in dimethyl sulfoxide and 0.5% hydrogen peroxide and measured using endpoint spectrometry.

### Determination of the total cell number and inflammatory cells

Using a previously described method [19], bronchoalveolar lavage was performed by instilling 0.9% NaCl containing 0.6 mmol/L EDTA in two separate 0.5 mL aliquots. The fluid was recovered by gentle suction and placed on ice for immediate processing. An aliquot of BALF was processed immediately for total and differential cell counts. The remainder of the lavage fluid was centrifuged and the supernatant was removed aseptically and stored in individual aliquots at −70°C. Total cell counts in BALF were determined using a haemocytometer. The number of inflammatory cells was calculated as the percentage of inflammatory cells multiplied by the total number of cells in the BALF sample. All analyses were performed in a blind fashion.

### Albumin concentration of BALF

The albumin content of the bronchoalveolar lavage supernatant was assessed using an ELISA kit for albumin (E91028Mu; Uscn Life Science Inc., Wuhan, China). Measurement of the absorbance at 450/540 nm was performed using a microplate reader (Infinite 200; Tecan Group, Zürich, Switzerland).

### Measurement of pulmonary oedema

Total lung water content, a quantification of pulmonary oedema, was measured as previously described [20]. The left lung was isolated for determination of total lung water content. The lung was weighed using an automatic electric balance (Sartorius, Goettingen, Germany) and was placed in an oven at 80°C for 48 h before being weighed again to obtain its dry weight. Total lung water content was calculated as follows: total lung water content = (wet lung weigh − dry lung weight)/(dry lung weight).

### Measurement of intracellular ROS

ROS was measured using a previously described method [21]. BAL cells were washed with PBS. To measure intracellular ROS, cells were incubated for 10 min at room temperature with PBS containing 3.3 μM 2′,7′-dichlorofluorescein (DCF) diacetate (Molecular Probes, Eugene, OR, USA) to label intracellular ROS. We performed fluorescence-activated cell sorting analysis with DCF stained cells (1 × 104 cells) in BALF to measure ROS levels using a FACSCalibur (BD Biosciences, San Jose, CA, USA). The data were analysed using CellQuest Pro (BD Biosciences).

### Measurement of glutathione and oxidised glutathione in lung tissues

Lung tissues were homogenised with 10 mL of ice-cold lysis buffer (50 mM phosphate buffer containing 1 mM EDTA) per gram of tissue. After centrifugation at 10,000 × *g* for 15 min at 4°C, the supernatant was removed, deproteinated, and stored at −20°C until further analyses. Total glutathione and oxidised glutathione levels were determined using a glutathione assay kit (Cayman Chemical Co., Ann Arbor, MI, USA), according to the manufacturer’s protocol.

### Determination of the total lung collagen content

The total lung collagen content was determined using the Sircol Collagen Assay Kit (Biocolor Ltd., Belfast, Northern Ireland) according to the manufacturer’s protocol.

### Lung histopathology

Lung tissues were fixed in 4% paraformaldehyde, embedded in paraffin, and cut into 5-μm thick sections. Sections were stained with hematoxylin and eosin, and images were taken with a Nikon Eclipse E800 microscope (200 ×). For the lung injury score, images were evaluated by an investigator who was blinded to the identity of the slides, as previously described [6]. Briefly, the extent of the pathological lesions was graded from 0 to 3 as shown in Table 1. The score for each animal was calculated by dividing the total score for the number of sections observed.

**Table 1:** **Acute lung injury pathology scoring criteria**

Score	Alveolar septa	Alveolar haemorrhage	Intra-alveolar fibrin	Intra-alveolar infiltrations per field
0	All are thin and delicate	No haemorrhage	No intra-alveolar fibrin	Less than 5 intra-alveolar cells
1	Congested alveolar septa in less than 1/3 of the field	Erythrocytes per alveolus in 1 to 5 alveoli	Fibrin strands in less than 1/3 of the field	5 to 10 intra-alveolar cells
2	Congested alveolar septa in 1/3 to 2/3 of the field	At least 5 erythrocytes per alveolus in 5 to 10 alveoli	Fibrin strands in 1/3 to 2/3 of the field	10 to 20 intra-alveolar cells
3	Congested alveolar septa in greater than 2/3 of the field	At least 5 erythrocytes per alveolus in more than 10 alveoli	Fibrin strands in greater than 2/3 of the field	More than 20 intra-alveolar cells

### Statistical analysis

Data are expressed as mean ± SEM, unless otherwise indicated. Data were analysed using ANOVA followed by the Newman-Keuls comparison. For two-group comparisons, an unpaired Student’s *t*-test was used (GraphPad Software, San Diego, CA, USA). A *P-*value <0.05 was considered statistically significant.

## Results

### Histology

**Figure 2:**
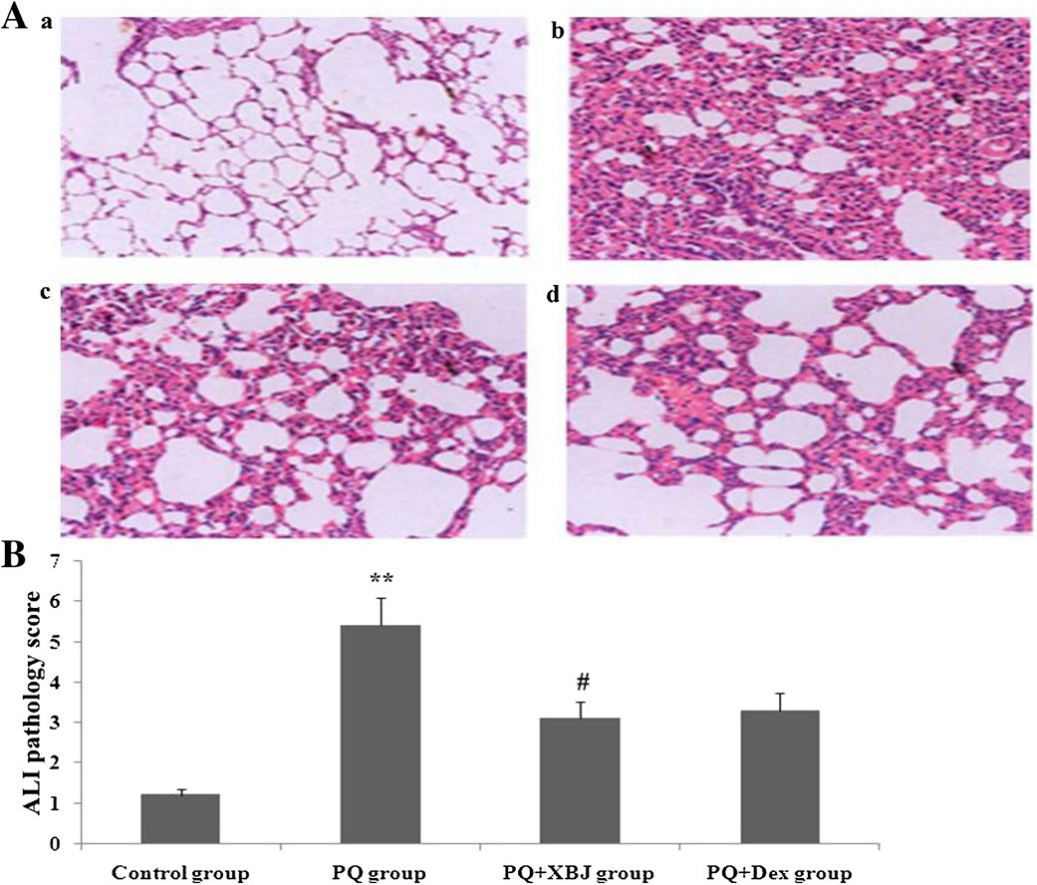
**Changes in lung pathology and lung injury score. A**: Representative lung pathological graphs (a: control group, b: paraquat group, c: Xuebijing + paraquat group, d: dexamethasone + paraquat group). **B**: Statistical analysis of lung injury score. Data are expressed as mean ± SEM. **P < 0.01 compared with control group; ^#^ *P* < 0.05 compared with paraquat group; *P* > 0.05 Xuebijing + paraquat group compared with dexamethasone + paraquat group.

To determine the effect of Xuebijing on histological lung injury, histopathological analysis was performed on sections stained with haematoxylin and eosin. As show the Figure 2, Histological analyses of lungs following paraquat exposure revealed that the capillaries in the lung tissue expanded and became congested by the significant increase in neutrophils. Additionally, lung septa thickened and did not show any improvement 48 h later, and lung injury score was markedly increased. Xuebijing + paraquat group rats also displayed moderate injury, but the severity was significantly ameliorated and lung injury score significantly decreased compared with the paraquat group, and compared with the dexamethasone + paraquat group, there were no significant differences.

### Xuebijing inhibited the expression of p-p38 MAPK, NF-κB65, p-IkB-α, HIF-1α and TGF-β1, and up-regulated IkB and Nrf2 in paraquat-induced lung tissue

**Figure 3:**
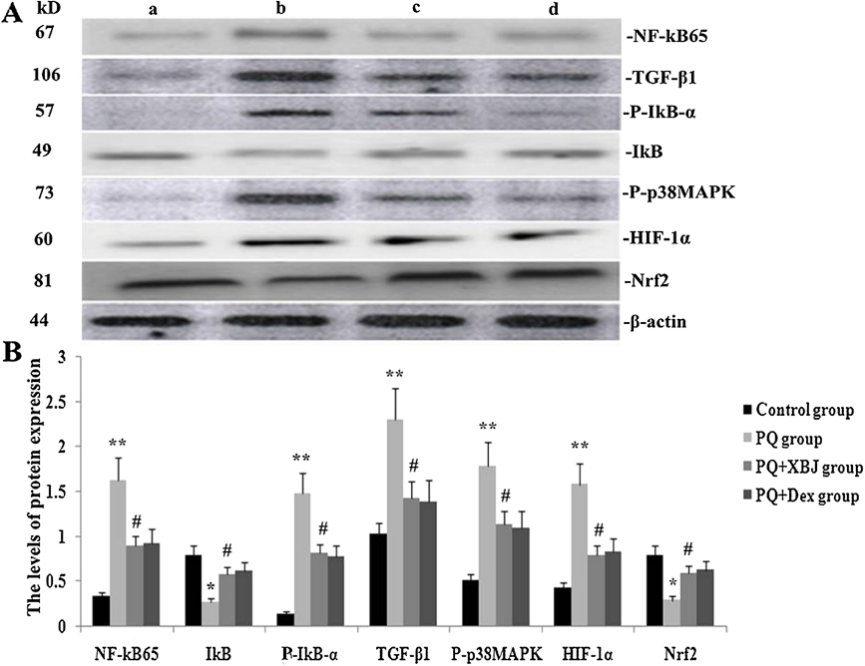
**Comparison of expression levels of p-p38 MAPK, NF-κB65, p-IκB-α, IkB, HIF-1α, Nrf2 and TGF-β1 using western blotting. A**: Representative graphs of protein expression of p-p38 MAPK, NF-κB65, p-IκB-α, IkB, HIF-1α, Nrf2 and TGF-β1 (a: control group, b: paraquat group, c: Xuebijing + paraquat group, d: dexamethasone + paraquat group). **B**: statistical analysis of protein expression of p-p38 MAPK, NF-κB65, p-IκB-α, IkB, HIF-1α, Nrf2 and TGF-β1. Data are expressed as mean ± SEM. **P < 0.01 compared with control group; ^#^ *P* < 0.05 compared with paraquat group; *P* > 0.05 Xuebijing + paraquat group compared with dexamethasone + paraquat group.

To assess the potential role of Xuebijing in paraquat-induced ALI, we determined the levels of p-p38 MAPK, NF-κB65, IkB, p-IκB-α, HIF-1α, Nrf2 and TGF-β1 proteins in lung tissue of paraquat-induced ALI rats using western blotting at 24 h after paraquat challenge (Figure 3). At 48 h after paraquat administration, lung p-p38 MAPK, NF-κB65, HIF-1α, p-IκB-α and TGF-β1 expression showed significant increases, and Nrf2 and IkB expression was significantly reduced. However, Xuebijing significantly attenuated the expression of p-p38 MAPK, NF-κB65, HIF-1α, p-IκB-α and TGF-β1, and up-regulated Nrf2 and IkB expression, and compared with the dexamethasone + paraquat group, there were no significant differences.

### Xuebijing inhibited the activation of NF-κB65 in paraquat-induced lung tissue

**Figure 4:**
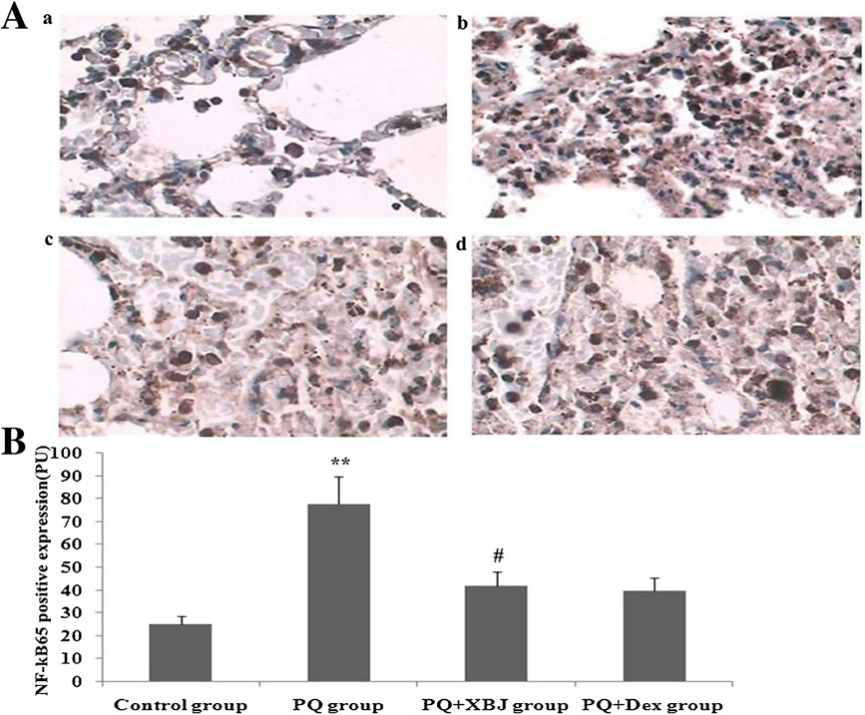
**Effect of Xuebijing on the activation of NF-κB65 in paraquat-induced lung tissue using immunohistochemical staining (×200). A**: Representative immunohistochemical-stained graphs of NF-κB65-positive expression (a: control group, b: paraquat group, c: Xuebijing + paraquat group, d: dexamethasone + paraquat group). **B**: Statistical analysis of NF-κB65-positive expression. Data are expressed as mean ± SEM. **P < 0.05 compared with control group; ^#^ *P* < 0.05 compared with paraquat group; *P* > 0.05 Xuebijing + paraquat group compared with dexamethasone + paraquat group.

Lung activation of NF-κB65 was very weak at 48 h in the control group (Figure 4). However, NF-κB65 expression was markedly increased in the paraquat and Xuebijing + paraquat groups. At 48 h after Xuebijing administration, activation of NF-κB65 was markedly inhibited in the Xuebijing + paraquat group, and compared with the dexamethasone + paraquat group, there were no significant differences.

### Xuebijing treatment attenuated systemic inflammation

**Figure 5:**
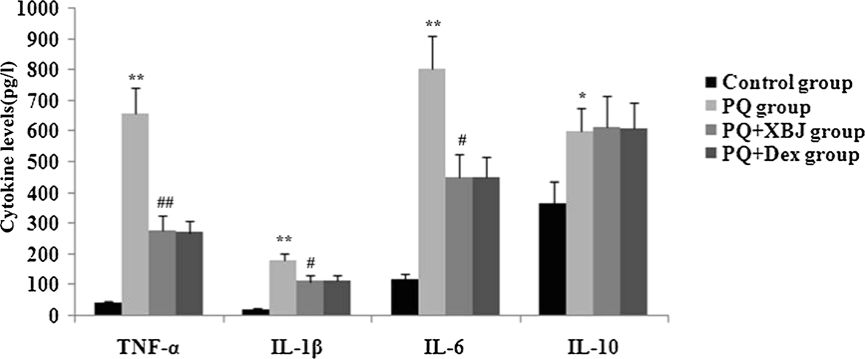
**Xuebijing treatment suppressed the production of inflammatory mediators.** The levels of TNF-α, IL-1β, IL-6, and IL-10 in serum were determined using ELISA. Data are expressed as mean ± SEM. **P < 0.01 compared with control group; ^#^ *P* < 0.05 and ^##^ *P* < 0.01 compared with paraquat group; *P >* 0.05 Xuebijing + paraquat group compared with dexamethasone + paraquat group.

Serum was collected at 48 h to evaluate the levels of TNF-α, IL-1β, IL-6, and IL-10. As shown the Figure 5, paraquat caused a significant acute systemic inflammatory response as demonstrated by the increased serum concentrations of the pro-inflammatory mediators TNF-α, IL-1β, and IL-6. The presence of Xuebijing reduced the increase in these three pro-inflammatory cytokines at 48 h, and compared with the dexamethasone + paraquat group, there were no significant differences. Paraquat also increased the serum concentration of the anti-inflammatory cytokine IL-10. This change in IL-10 concentration was not altered by intravenous administration of Xuebijing and dexamethasone.

### Xuebijing treatment prevented inflammatory cell infiltration in the lungs

**Figure 6:**
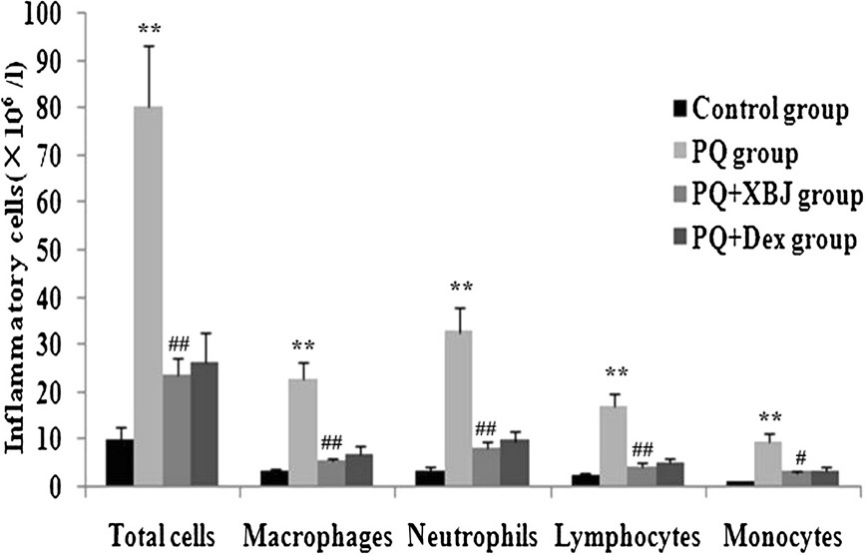
**Xuebijing treatment prevented inflammatory cell infiltration in paraquat-induced lung injury.** Inflammatory cells in BALF were measured. Data are expressed as mean ± SEM. **P < 0.01 compared with control group; ^#^ *P* < 0.05 and ^##^ *P* < 0.01 compared with paraquat group; P > 0.05 Xuebijing + paraquat group compared with dexamethasone + paraquat group.

To investigate the potential mechanism underlying the protective effect of Xuebijing on paraquat-induced ALI, we detected the total number of cells, neutrophils, macrophages, lymphocytes and monocytes in BALF from rats treated with paraquat with or without Xuebijing. As shown the Figure 6, paraquat caused a significant increase in inflammatory cell counts in BALF at 48 h. These increases were reduced in the Xuebijing + paraquat group, and compared with the dexamethasone + paraquat group, there were no significant differences.

### Xuebijing treatment ameliorated oxidative stress

**Figure 7:**
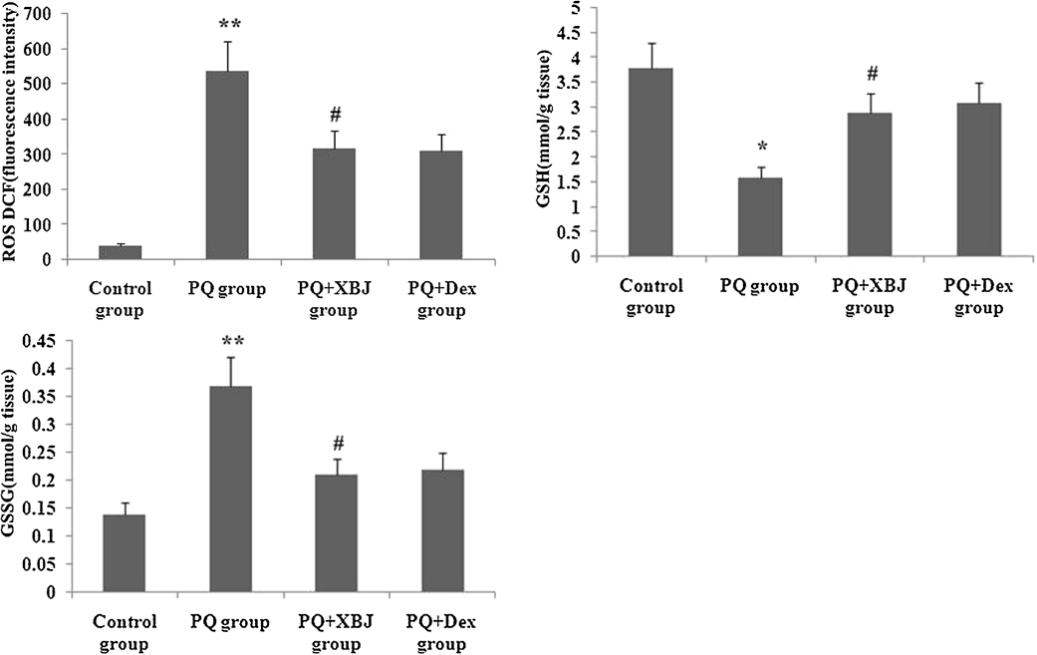
**Effect of Xuebijing treatment on ROS, glutathione and oxidised glutathione activities.** ROS, glutathione and oxidised glutathione activities were determined. Data are expressed as mean ± SEM. **P < 0.01 compared with control group; ^#^ *P* < 0.05 compared with paraquat group; *P* > 0.05 Xuebijing + paraquat group compared with dexamethasone + paraquat group.

To assay the effect of Xuebijing treatment on oxidative stress, ROS, glutathione and oxidised glutathione activities were determined. As shown in Figure 7, ROS and oxidised glutathione activities were significantly higher, and glutathione activity was markedly lower, at 48 h after paraquat administration. Xuebijing treatment significantly attenuated ROS and oxidised glutathione activities and elevated glutathione activity, and compared with the dexamethasone + paraquat group, there were no significant differences.

### Xuebijing treatment reduced the wet/dry weight ratio and BALF protein concentration

**Figure 8:**
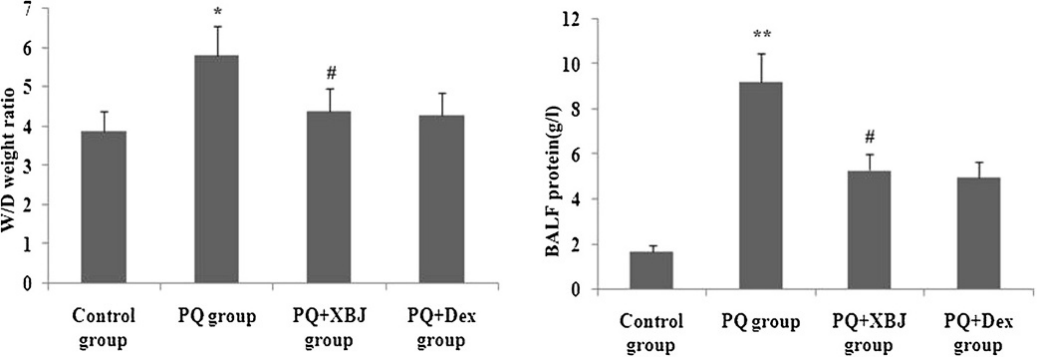
**Effects of Xuebijing treatment on the wet/dry weight ratio and BALF protein.** Data are expressed as mean ± SEM. *P < 0.05; **P < 0.01 compared with control group; ^#^ *P* < 0.05 and ^##^ *P* < 0.01 compared with paraquat group; *P* > 0.05 Xuebijing + paraquat group compared with dexamethasone + paraquat group.

The lung wet-dry weight ratio was significantly higher at 48 h after paraquat administration. Xuebijing treatment significantly attenuated this change (Figure 4A). Additionally, with regard to endothelial and epithelial permeability, the BALF protein concentration quickly increased after paraquat injection. The paraquat + Xuebijing group also exhibited significantly lower protein concentrations, and compared with the dexamethasone + paraquat group, there were no significant differences (Figure 8).

### Xuebijing treatment reduced TGF-β1 and PIIIP expression

**Figure 9:**
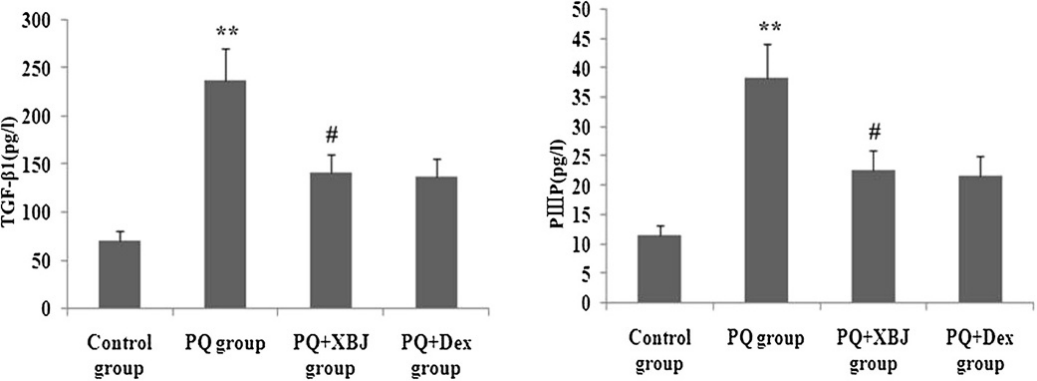
**Xuebijing treatment inhibited the generation of TGF-β1 and PIIIP.** Levels of TGF-β1 and PIIIP in serum were determined using ELISA. Data are expressed as mean ± SEM. **P < 0.01 compared with control group; ^#^ *P* < 0.05 and ^##^ *P* < 0.01 compared with paraquat group; *P* > 0.05 Xuebijing + paraquat group compared with dexamethasone + paraquat group.

Concentrations of serum TGF-β1 and PIIIP were markedly increased at 48 h after paraquat administration in the paraquat group and Xuebijing + paraquat group; however, the increase in TGF-β1 and PIIIP were suppressed by Xuebijing in paraquat-induced rats, and compared with the dexamethasone + paraquat group, there were no significant differences (Figure 9).

## Discussion

Intentional oral ingestion of even small amounts of paraquat can cause severe and irreversible systemic damage refractory to any known treatment [5]. Upon ingestion, paraquat is rapidly distributed to most organs in the body, but the highest concentrations are found in the kidneys and lungs [7]. Paraquat accumulation in the lungs is facilitated by type I and II alveolar epithelial cells via the polyamine uptake pathway. Subsequent redox cycling and free radical generation initiate a neutrophil-mediated inflammatory response in the lungs [10].

In the current study, paraquat was found to stimulate the infiltration of inflammatory cells into the interstitial and alveolar spaces, and to increase the generation of pro-inflammatory mediators, such as IL-6, TNF-α and IL-1β1. Pathological findings demonstrated disrupted alveolar epithelial cells, haemorrhage, oedema, and infiltration of inflammatory cells into lung tissue. Treatment with Xuebijing suppressed the infiltration of inflammatory cells into the interstitial and alveolar spaces, reduced increases in the generation of pro-inflammatory mediators, and extenuated lung injury.

Glucocorticoids, such as dexamethasone, are potent anti-inflammatory drugs frequently prescribed for the treatment of various inflammatory diseases, including asthma, chronic obstructive pulmonary disease, and acute respiratory distress syndrome [22]. In addition to these chronic inflammatory diseases, glucocorticoids have also been used for the treatment of severe sepsis and septic shock in patients in the intensive care unit [23]. Clinical trials have indicated that low dose glucocorticoids alleviate the systemic inflammatory response, reduce the duration of shock, and favourably affect survival in patients with septic shock [22]. In animal models of endotoxic shock, prophylactic treatment with dexamethasone attenuates the production of inflammatory cytokines including TNF-α and IL-1β, and prevents shock and mortality [24]. Previous studies using prolonged administration of corticosteroids, such as methylprednisolone or dexamethasone, initiated before or simultaneously with bleomycin reduced pulmonary inflammation, lung injury, and collagen deposition in this model [25–27].

Building on this previous research, and to investigate Xuebijing’s clinical effects, we established a dexamethasone group as a positive control group. We found that dexamethasone was as effective as Xuebijing at blocking lung p-p38 MAPK, NF-κB65, HIF-1α, p-IκB-α and TGF-β1 expression, increasing Nrf2 expression, inhibiting the inflammatory response and oxidative stress, and extenuating paraquat-induced lung injury.

The present study investigated the effect of Xuebijing on paraquat-induced extra-pulmonary ALI. The experimental model was previously validated as a model of ALI [28], and there is evidence that the metabolic processes, immuno-inflammatory features and histological lung damage in acute paraquat intoxication are similar in humans and rats [29, 30]. The dose of paraquat used (30 mg/kg of body weight. i.p.) may be classified as moderate exposure to paraquat (60% of the LD50) in rats (LD50 = 50 mg/kg) and high exposure in humans, considering a human LD50 of 35 mg/kg [31]. This dose was sufficient to induce an inflammatory response with increased cell migration and resulted in lung injury, as demonstrated by lung oedema, haemorrhage, alveolar obstruction and collapse. Results from the study showed that Xuebijing treatment markedly inhibited the secretion of inflammatory mediators, reduced cell migration, attenuated lung oedema, haemorrhage, alveolar obstruction and collapse.

The inflammatory reaction plays an important role in ALI in patients following paraquat poisoning, with the release of pro-inflammatory factors resulting in systemic inflammatory response syndrome, as well as the release of anti-inflammatory factors [4]. Clinically, the expression of inflammatory cytokines in the blood is significantly elevated in patients with paraquat poisoning [14].

A limited number of transcription factors regulate the inflammatory pathways, of which the most important transcriptional regulator is NF-κB [32]. Following activation by a wide array of mediators, including cytokines, bacterial toxins, or oxidative stress, the signal transduction cascade is initiated, and activated NF-κB can translocate to the nucleus and bind to the promoters of pro-inflammatory genes, leading to enhanced gene expression and amplification of the inflammatory response. IκB-α (nuclear factor of kappa light polypeptide gene enhancer in B-cells inhibitor, alpha) is one member of a family of cellular proteins that function to inhibit NF-κB transcription factor. IκB-α inhibits NF-κB by masking the nuclear localisation signals of NF-κB proteins and keeping them sequestered in an inactive state in the cytoplasm [33]. In addition, IκB-α blocks the ability of NF-κB transcription factor to bind to DNA, which is required for NF-κB’s proper functioning [34]. As the expression of these pro-inflammatory mediators is modulated by NF-κB, blocking NF-κB transcriptional activity may be an important target for treating inflammatory diseases.

Paraquat stimulation elicits a cascade which increases p-IκB-α expression and reduces IkB expression, resulted in the activation of NF-κB and the enhanced secretion of pro-inflammatory mediators, which results in lung injury.

The MAPK signalling pathway in macrophages is one of the most extensively investigated intracellular signalling cascades involved in lipopolysaccharide-induced pro-inflammatory responses [35], which are classified into at least three components: extracellular signal-regulated kinases 1/2, c-Jun N-terminal kinase, and p38 MAPK, and which have been implicated in the release of immune-related cytotoxic factors and pro-inflammatory cytokines [35]. p38 MAPK is a key mediator of cellular stressors such as inflammation and apoptosis. Both *in vitro* and *in vivo* studies have shown that p38 MAPK regulates the production of pro-inflammatory cytokines IL-6 and TNF-α by increasing cytokine release or messenger RNA transcription [36]. Earlier reports revealed that inhibitors of p38 MAPK (SB203580) reduce lipopolysaccharide-induced pro-inflammatory protein levels [37]. Numerous basic and clinical studies have demonstrated that overexpression of TNF-α and IL-1ß can induce lung injury and that cytokine production is associated with activation of the p38 MAPK pathway [35–38]. Results from our study indicate that paraquat increased p-p38 MAPK expression and further stimulated expression of TNF-α and IL-1ß. However, the increase in p-p38 MAPK expression was inhibited by Xuebijing which inhibited the expression of TNF-α and IL-1ß.

Several lines of evidence suggest that MAPK can participate in the activation of NF-κB in the cytoplasm as well as in the modulation of its transactivation potential in the nucleus. As the expression of these pro-inflammatory mediators is modulated by NF-κB, blocking NF-κB transcriptional activity may be an important target for treating inflammatory diseases. Our findings suggest that the inhibition of MAPK pathways by Xuebijing may be a major mechanism underlying the attenuation of paraquat-induced NF-κB transcriptional activity.

Oxidative stress is a sign of inflammation. Previous studies on various lung inflammation diseases have confirmed that oxidative stress and oxidative damage are closely related to the development and severity of ALI/acute respiratory distress syndrome [39]. An imbalance between ROS and the antioxidant defence system results in oxidative stress, which is closely linked to the pathogenesis of acute and chronic lung injury [39]. Increased levels of ROS can cause direct tissue injury and promote inflammatory responses via the regulation of diverse pro-inflammatory mediators in the lungs. The response of a cell to excessive ROS involves the activation of multiple signalling pathways, which can cause transcriptional changes and, consequently, exhibit a variety of activities [40, 41]. NF-κB is one of the major transcription factors activated in the lung during oxidative stress, leading to an up-regulation of numerous pro-inflammatory genes, such as T-helper 2 cytokines, which can affect lung injury [42–44]. ROS also attenuates the transcriptional activity of nuclear factor E2-related factor 2 (Nrf2) and stimulates hypoxia-inducible factor (HIF)-1α expression, resulting in a greater expression of their target genes [45–47]. Some studies have also indicated that ROS functions as a signalling molecule and can stimulate HIF-1α protein synthesis via the activation of the PI3K/AKT and p42/p44 MAPK pathways. Furthermore, ROS may also have the potential to interfere with prolyl hydroxylase activity to regulate HIF-1α expression. Glutathione synthesised from cysteine is a vital protective antioxidant against oxidative stress. Our research found that paraquat down-regulated Nrf2 expression via increasing NF-κB, HIF-1α and MAPK activity, increased ROS activity, and inhibited glutathione activity. Expectedly, Xuebijing treatment up-regulated Nrf2 expression via the reduction of the increase in NF-κB, HIF-1α and MAPK activity, markedly enhanced glutathione activity, and improved paraquat-induced lung injury.

TGF-β1 is known to enhance the fibrotic process by enhancing fibroblast growth and collagen production as well as promoting the differentiation of fibroblasts into myofibroblasts, which secrete collagen and other extracellular matrix components [48]. In addition, TGF-β1 can affect several signal transduction pathways in a Smad-independent manner, such as MAPK, including extracellular signal-related protein kinase, p38 MAPK, and c-Jun N-terminal kinase [49].We also examined TGF-β1 expression in the present paraquat-induced mouse model. Consistent with these previous findings, our results showed that expression of TGF-β1 and p-p38 MAPK increased after paraquat challenge, and administration of Xuebijing in paraquat-induced rats decreased the increased expression of TGF-β1 by blocking the p38 MAPK pathway.

PIIIP is a crucial component of type III collagen synthesis and is the major collagenous factor in fibrosis [50]. Current perspectives suggest that the increased synthesis of collagen type I, III, and V may play an important role in the pathophysiological mechanism, resulting in intestinal fibrosis [51]. Furthermore, the increased synthesis of collagen, namely, an increase in procollagen type III, has been well documented in fibrotic processes involving other organs, such as the liver, pancreas and lung [52]. In this study, we found that paraquat significantly enhanced type III collagen synthesis. However, Xuebijing markedly reduced the production of PIIIP by inhibiting TGF-β1 and p38 MAPK expression.

Vascular leakage in multiple organs is a characteristic pathological change in paraquat-induced lung injury [53]. In the present study, we evaluated the wet/dry ratio of the lung and found that Xuebijing treatment attenuated the development of pulmonary oedema, as determined by the significant decrease in lung wet/dry ratio.

As another index of ALI caused by paraquat administration, we measured the total protein concentration in BALF, which indicates epithelial permeability and pulmonary oedema [54]. As expected, paraquat treatment was found to cause a significant increase in BALF protein concentration. Paraquat-induced increases in total protein in BALF were inhibited by Xuebijing through regulating the p38 MAPK pathway.

Xuebijing is a plant-derived Traditional Chinese Medicine that has been successfully applied to treat inflammation-associated diseases, such as sepsis and severe pneumonia [16]. Some studies have revealed its specific effects on various immunomodulatory factors and processes. Xuebijing treatment of peripheral blood mononuclear cells stimulated by exposure to purified bacterial endotoxin lipopolysaccharide has resulted in decreases in tissue factor and protease-activated receptor expression [17]. Severe pneumonia patients treated with Xuebijing have shown decreased secretion of TNF-α, IL-6 and IL-8 [55], similarly to patients with sepsis. In addition, Xuebijing treatment, which has been clinically administered, is an intravenous preparation consisting of 32 traditional Chinese medicines. The principal components include Flos Carthami, Radix Paeoniae Rubra, Rhizoma Chuanxiong, Radix Salviae miltiorrhizae [56], Radix Angelica sinensis and Herba houttuyniae. Intravenous Xuebijing treatment is widely used throughout China and has shown clinical success in the treatment of systemic inflammatory response syndrome and multiple organ dysfunction syndrome [56]. The active ingredients of the preparation were recently determined using mass spectrophotometry and included safflor yellow A, ligustrazine, tanshinol, ferulaic acid, and peoniflorin. Ferulaic acid has been previously described for its ability to eliminate oxygen free radicals and to prevent pulmonary fibrosis [17, 18].

This study confirmed that treatment with Xuebijing reduced the expression of p38 MAPK and stimulated a reduction in NF-κB65 activity. These findings were evident in the paraquat + Xuebijing group. In addition, reducing the levels of IL-6, IL-1β and TNF-α attenuated the morphological lesions of lung parenchyma; however, the best results were observed in the paraquat + Xuebijing group. In this group, lung oedema was reduced, and there was a slight thickening of the alveolar septum, reduction in inflammatory cell migration and a greater area of free alveolar surface compared with the paraquat groups.

## Conclusions

**Figure 10:**
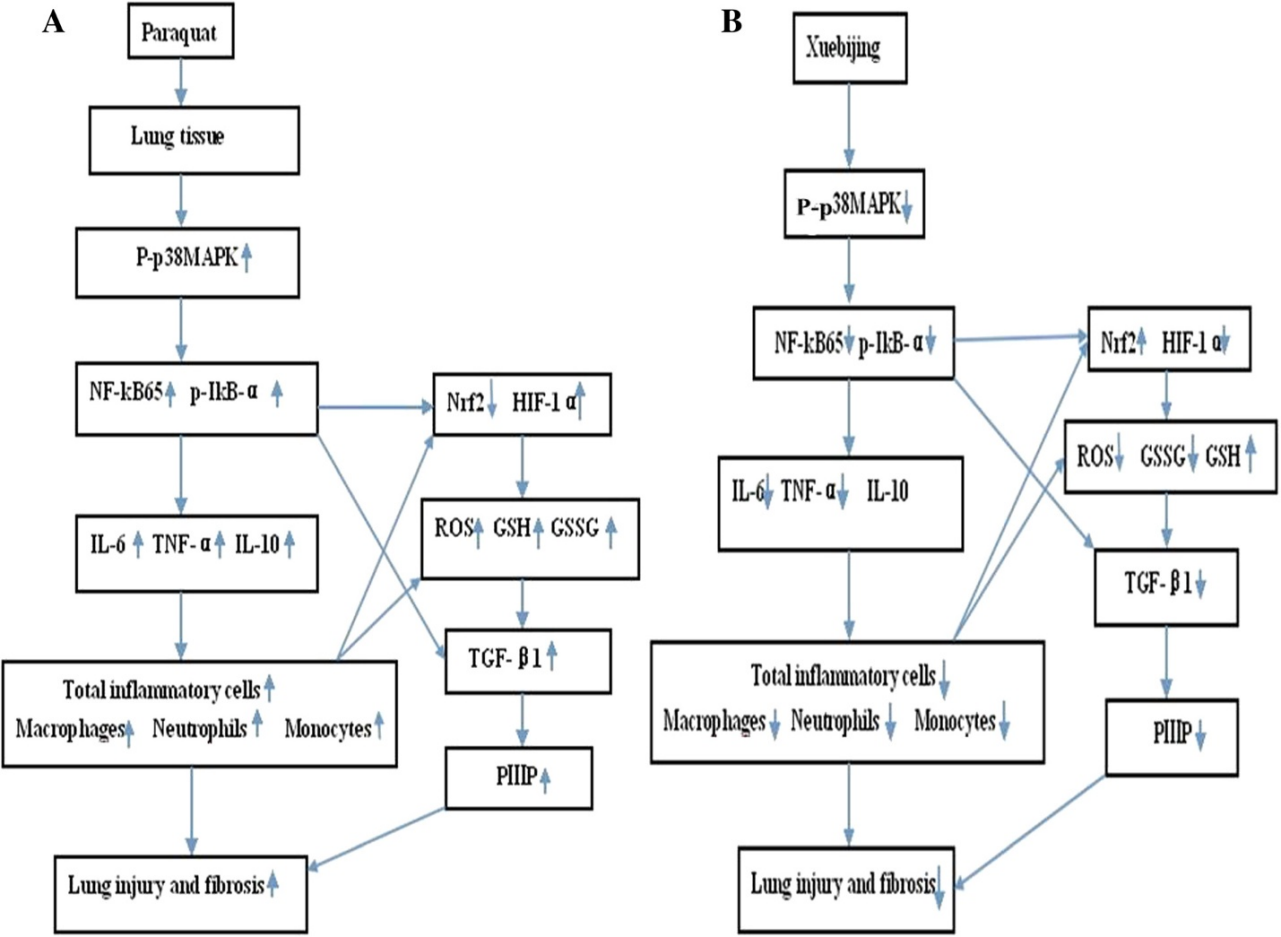
**Charts of the mechanisms of the effects of paraquat and Xuebijing on lung injury. A**: Chart of the mechanisms of paraquat-induced lung injury. **B**: Chart of the mechanisms of the effects of Xuebijing on paraquat-induced lung injury.

This study demonstrated that Xuebijing treatment reduced paraquat-induced extra pulmonary ALI in Sprague Dawley rats. The results indicate the effect of its inhibitory activities on p38 MAPK and NF-κB65, the secretion of pro-inflammatory mediators, oxidative stress response, the attenuation of lung permeability leakage and the reduction of morphological damage in lung parenchyma (Figure 10). Further pharmacological evaluations are essential to elucidate the detailed mechanism of the activity of Xuebijing, which may provide insight on its potential to prevent and treat extrapulmonary ALI.

## Abbreviations

ALI: Acute lung injury
TNF-α: Tumour necrosis factor-alpha
IL-6: Interleukin-6
TGF-β: transforming growth factor-β
MAPK: mitogen-activated protein kinase
Nrf2: E2-related factor 2
HIF-1α: Hypoxia-inducible factor-1α
NF-κB: Nuclear factor-κB
PIIIP: Collagen III
ROS: Reactive oxygen species
BALF: Bronchoalveolar lavage fluid.
